# Immobilization of Cr^3+^, Cd^2+^, and Pb^2+^ added to calcareous soil amended with composted agro-industrial residues

**DOI:** 10.1038/s41598-023-35358-3

**Published:** 2023-05-20

**Authors:** Mai Khedr, Mohamed Emran, Maria Gispert, Mohamed Rashad

**Affiliations:** 1 Land and Water Technologies Department, Arid Lands Cultivation Research Institute (ALCRI), City of Scientific Research and Technological Applications (SRTA-City), New Borg El-Arab City, 21934 Alexandria, Egypt; 2 Department of Chemical Engineering, Agriculture and Food Technology, University of Girona, C Maria Aurèlia Capmany, 61, Campus Montilivi, 17003 Girona, Spain

**Keywords:** Environmental chemistry, Environmental impact

## Abstract

The bioavailability of trace metals in soils poses a major threat to the environment, especially with massive mineral fertilizers added to increase plant yield. A plot experiment was conducted for the effectiveness evaluation of compost and vermicompost, recycled from agro-industrial wastes, in immobilizing chromium, cadmium, and lead added to calcareous soil (artificially contaminated). Moreover, immobilization efficiency was compared to the natural occurrence of these metals in the soil without metal addition (uncontaminated soil). In both soils, amendments and mineral fertilizers were applied at three different levels alone and combined to each other. The experimental design was arranged in factorial complete randomized blocks using contamination, organic and mineral fertilizer levels, and their combination as categorical factors. The distribution of metal fractions and their bioavailability in soils and bioaccumulation in wheat grains were evaluated. Soil alkalinity, the contents of soil organic carbon and nitrogen, available phosphorus, and soil micronutrients were significantly improved under vermicompost and compost compared to mineral fertilizer and control. Vermicompost was more effective than compost in reducing metals bioavailability in contaminated soils by increasing the immobilized organic fractions, but it regressed when combined with mineral fertilizers. The bioavailability of the naturally occurring metal levels in uncontaminated soil did not change significantly compared to contaminated soil. Likewise, wheat yield, plant biomass, and nutrient enrichment in wheat grains improved due to enhanced soil nutrient availability. These composted agro-industrial residues, by-products from food industries, can be classified as environmentally-friendly soil amendments for their great potential to enrich soil nutrients, reduce mineral fertilizer addition, enhance plant growth, and stabilize Cr, Cd, and Pb in contaminated calcareous soils under wheat plants.

## Introduction

The agro-industrial residue is defined as the many different wastes generated by the food and agricultural industries^1^. In recent years, environmental problems have strengthened their importance and increased interest in the efficient use of waste from various agricultural industries^2^ As by-products, they should be identified as residues, not wastes, due to their nutritional value that should not be overlooked with no contamination risks. Their management plays a crucial role in the conservation of natural resources and represents an environmental and economic concern due to the huge quantities generated and their pollutant levels, thus more research is needed to reduce their discharge to the environment and management costs^3^. Moreover, they are rich in nutrients and bioactive components and are of interest as raw materials for the formation of natural fertilizers and biofuel^1^. Currently, they are used as animal feed or composting, and most of them are commonly used as fuel in traditional low-performance stoves or directly burned contributing to environmental pollution. In front of this, more friendly techniques should be experimented with to control their decomposition and reduce environmental risks. Composting and vermicomposting are examples of controlled decomposition techniques that have been proven as beneficial alternatives to be used as fertilizers^4–6^. Their decomposition in the soil allows the formation of strong polymerizing particles forming more stable organic forms^7^. However, the singularity of waste types or mixtures may go through different decomposition processes thus producing diverse end-products differing in their chemical and biochemical properties^5^.

The content of transition metals in agro-industrial residues has been often detected at rates below the allowed limits though they can accumulate in crops and related residues. Consequently, they may return to the soil as recycled fertilizers. However, whilst essential elements such as iron (Fe), manganese (Mn), copper (Cu), zinc (Zn), and nickel (Ni) are beneficial to plants as micronutrients^8^, non-essential metals such as cadmium (Cd), lead (Pb), arsenic (As), mercury (Hg), and chromium (Cr) naturally occur in soil may be highly detrimental for crops^9,10^. This study focused to investigate the bioavailability and immobilization of Cr, Cd, and Pb as naturally exist in the Egyptian agricultural lands under low irrigation water-quality. These metal forms are typically existed as cationic (i.e., Cd and Pb) and anionic (Cr) species as they complex with inorganic soil constituents (carbonates, sulfates, hydroxides, sulfides) and oxygen to form either precipitated or charged (positively or negatively) complexes (CrO_4_^2−^)^11^. The toxicity of non-essential metals may increase at high concentrations thus posing a high concern for environmental quality and human health, because of their resistance to microbial degradation^12^. The stabilized organic matter amended to the soil after composting of these residues may play a relevant role in the immobilization of non-essential elements forming chelated organometallic complexes. Due to metal complexation by organic bonds, their mobility and availability may be substantially decreased^13^. However, the chemistry of chelating agents and metals is complex^14^ and may be influenced by soil factors such as salinity, pH, redox potential, and clay minerals^13,15^, the latter being important in metal immobilization by adsorption mechanisms^16^. Besides metals adsorption mechanisms attributed to clay and organic matter, metals precipitation may occur in carbonate and oxide-rich soils under alkaline conditions. Metal fractions could be precipitated in carbonate forms in alkaline calcareous soils depending on the activity of calcium carbonate. However, sulfates may also release metal ions into the soil due to bridging effect of sulfate with calcium depending on soil pH and redox conditions^17,18^.

This work aimed (i) to amend poorly structured calcareous soils with compost and vermicompost derived from agro-industrial residues as organic fertilizers to improve soil properties and crop quality, ii) to study their effects on the mobility of added chromium, cadmium, and lead and their uptake by wheat grains, and iii) to evaluate their bioavailability and immobilization in the artificially contaminated soil compared to their baseline level in the uncontaminated soil at field scale.

## Results

### General soil characteristics

Soil texture is classified as sandy clay loam (60.59% sand, 12.82% silt, and 26.59% clay). According to the USDA classification of Egypt’s Nile Delta soils, the soil was classified as Typic Calcitorrerts^19^ with 35.02±1.21% (n=3) CaCO_3_ (detected using the Scheibler method, Royal Eijkelkamp calcimeter, Giesbeek, Netherlands). The general soil chemical characteristics are presented (Supplementary Table S1). Using Tukey’s HSD test, all parameters varied significantly (*P*<0.05) within and between the studied treatments in both uncontaminated (small superscript letters) and contaminated (capital superscript letters) soils. Moderate alkaline pH values were observed and decreased significantly under compost and vermicompost in both soils (uncontaminated and contaminated). Soil pH, SOC, TN, and available P and K significantly improved along the organic treatments under compost and vermicompost^20^. In both soils, slight salinity values were observed but increased significantly along the organic and mineral sub-treatments due to the chemical behavior of available trace elements^21^. Soluble sodium decreased significantly by 13% and 22% under compost and vermicompost, respectively compared to NPK (nitrogen, phosphorus, and potassium) and control sub-treatments in both soils^22^. Other soluble cations significantly improved along the organic treatments in both soils. Their low contents were attributed to the increased alkalinity, salinity, and CaCO_3_ contents^23^. The SAR decreased significantly along the organic treatments in both soils.

A highly significant negative correlation was only observed between the H^+^ concentration relative to the soil pH values and EC in the uncontaminated soil (r=−0.729, *P*<0.001, n=27). SOC increased by 263%, 157%, 126% in Cp, Cp+NPK50, and Cp+NPK100 and by 204%, 119%, and 94% in Vp, Vp+NPK50, and Vp+NPK100 in uncontaminated soil, respectively. In contaminated soil, SOC increased by 621%, 44%, and 63% in Cp, Cp+NPK50, and Cp+NPK100 and by 376%, 23%, and 20% in Vp, Vp+NPK50, and Vp+NPK100, respectively. TN increased by 77%, 67%, and 105% in Cp, Cp+NPK50, and Cp+NPK100 and by 106%, 144%, and 125% in Vp, Vp+NPK50, and Vp+NPK100 in uncontaminated soil, respectively. In contaminated soil, TN increased by 109%, 91%, and 76% in Cp, Cp+NPK50, and Cp+NPK100 and by 312%, 290%, and 191% in Vp, Vp+NPK50, and Vp+NPK100, respectively. Available phosphorus (P_AV_) increased by 13%, 11%, and 11% under Cp, Cp+NPK50, and Cp+NPK100 in both soils. It was also increased by 20%, 33%, and 36% under Vp, Vp+NPK50, and Vp+NPK100, respectively, in both soils. Available potassium (K_AV_) increased by 53%, 23%, and 17% in Cp, Cp+NPK50, and Cp+NPK100 and by 145%, 51%, and 23% in Vp, Vp+NPK50, and Vp+NPK100 in uncontaminated soil, respectively. It was also increased by 39%, 64%, and 53% in Cp, Cp+NPK50, and Cp+NPK100 and by 139%, 115%, and 91% in Vp, Vp+NPK50, and Vp+NPK100 in contaminated soil, respectively (Supplementary Table S1). Soil pH negatively correlated with SOC (r= −0.797, *P*<0.01; r= −0.876, *P*<0.01), TN (r=−0.497, *P*<0.01; r= −0.464, *P*<0.01), P_AV_ (r= −0.442, *P*<0.05; r= −0.483, *P*<0.01), and available K^+^ (r= −0.434, *P*<0.05; r= −0.548, *P*<0.01) in uncontaminated and contaminated soils, respectively. EC increased significantly with SOC (r=0.828, *P*<0.01; r=0.705, *P*<0.01), TN (r=0.827, *P*<0.01; r=0.764, *P*<0.01), P_AV_ (r=0.764, *P*<0.01; r=0.764, *P*<0.01), and K^+^ (r=0.697, *P*<0.01; r=0.724, *P*<0.01) in uncontaminated and contaminated soil, respectively.

### Distribution of metal fractions in soil

Metal contents were rationally higher in contaminated than in uncontaminated soils. Using Tukey´s HSD test, all metal fractions varied significantly (*P*<0.05) within and between the studied treatments in uncontaminated and contaminated soils as indicated by the different letters labeled above the distributed fractions.

### Distribution of chromium fractions in soil

**Figure 1:**
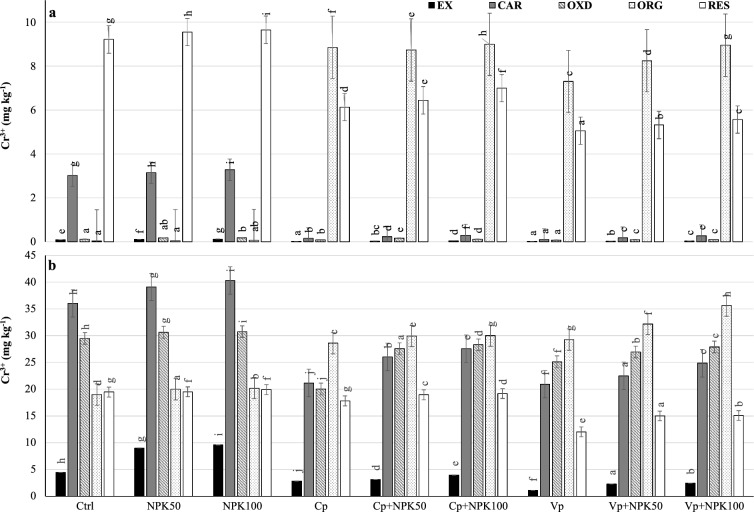
Distribution of Cr fractions in uncontaminated (**a**) and contaminated (**b**) soils. *Cd*_*EX*_ Exchangeable fraction, *Cd*_*CAR*_ Carbonate associated fraction, *Cd*_*OXD*_ Fe–Mn oxides bound fraction, *Cd*_*ORG*_ Organically bound fraction, *Cd*_*RES*_ Residual fraction, *Ctrl* Control, *NPK50* 50% of NPK dose, *NPK100* 100% of NPK dose, *Cp* Compost, *Cp+NPK50* Compost combined with 50% NPK, *Cp+NPK100* Compost combined with 100% NPK, *Vp* Vermicompost, *Vp+NPK50* Vermicompost combined with 50% NPK, *Vp+NPK100* Vermicompost combined with 100% NPK.

The exchangeable (Cr_EX_) and carbonate (Cr_CAR_) fractions are considered the primary and the additionally available fractions, respectively^9,13^. In uncontaminated soil (Fig. 1A), the Cr_EX_ and Cr_CAR_ fractions decreased significantly by 70–80% and 90–95%, respectively, under organic sub-treatments compared to control sub-treatments. Likewise, the Cr_OXD_ fraction decreased significantly by 30–40% under organic sub-treatments compared to control sub-treatments. However, the Cr_ORG_ fraction (immobilized fraction corresponds to metal associated with the organic fraction of the soil) extremely increased (135–250 times) in organic sub-treatments compared to control sub-treatments (Fig. 1A).. This fraction decreased by 48% at Cp+NPK50 and 27% at Cp+NPK100 compared to Cp and by 37% at Vp+NPK50 and 17% at Vp+NPK100 compared to Vp, confirming its reduction due to NPK addition. As a result, the Cr_RES_ fraction decreased significantly by 45–30% under organic sub-treatments compared to control sub-treatments.

In contaminated soil (Fig. 1B), the Cr_EX_ fraction decreased significantly by 30–75% and 30–40% under vermicompost and compost, respectively compared to control sub-treatments. Likewise, the Cr_OXD_ fraction decreased significantly by 15–10% and 30–10% under vermicompost and compost, respectively, compared to control sub-treatments. The Cr_ORG_ fraction significantly increased at these high Cr contents by 45–75% under organic sub-treatments compared to control sub-treatments (Fig. 1B). However, the NPK additions deteriorated its formation under organic sub-treatments with high efficiency under vermicompost. As a result, the Cr_RES_ fraction decreased significantly in organic sub-treatments compared to control sub-treatments.

### Distribution of cadmium fractions in soil

**Figure 2:**
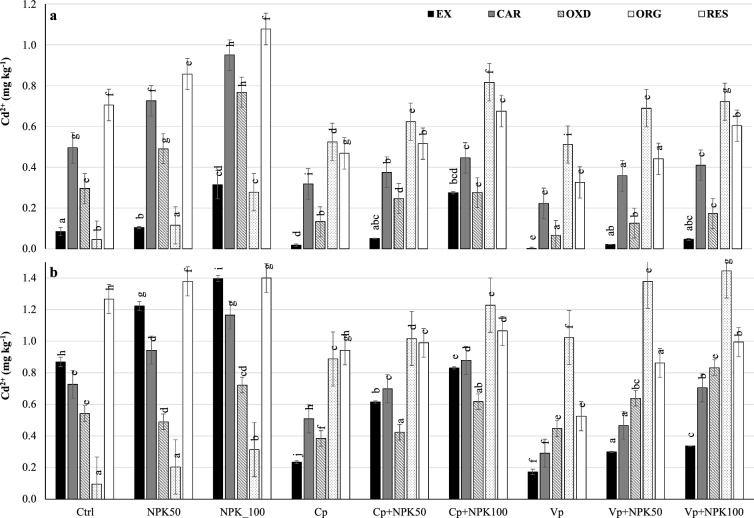
Distribution of Cd fractions in uncontaminated (**a**) and contaminated (**b**) soils. *Cd*_*EX*_ Exchangeable fraction, *Cd*_*CAR*_ Carbonate associated fraction, *Cd*_*OXD*_ Fe–Mn oxides bound fraction, *Cd*_*ORG*_ Organically bound fraction, *Cd*_*RES*_ Residual fraction, *Ctrl* Control, *NPK50* 50% of NPK dose, *NPK100* 100% of NPK dose, *Cp* Compost, *Cp+NPK50* Compost combined with 50% NPK, *Cp+NPK100* Compost combined with 100% NPK, *Vp* Vermicompost, *Vp+NPK50* Vermicompost combined with 50% NPK, *Vp+NPK100* Vermicompost combined with 100% NPK.

In uncontaminated soil (Fig. 2A), the Cd_EX_ fraction decreased significantly by 80–100% under vermicompost and by 50–75% under compost compared to control sub-treatments. The carbonate bound fraction decreased significantly by 50–55% under vermicompost and by 36–50% under compost compared to control sub-treatments (Fig. 2A). Likewise, the Cd_OXD_ fraction decreased significantly by 70–80% under vermicompost and by 50–55% under compost compared to control sub-treatments. However, the organic-bound fraction enormously increased 10-times when compost and vermicompost were applied alone but increased 5-times and one-time when combined with 50% and 100% NPK, respectively, confirming the increasing degradation or reduction of organic-metal complexes caused by the NPK increasing rates. As a result, the residual fraction decreased significantly by 55–35% under organic sub-treatments compared to the control (Fig. 2A).

In contaminated soil (Fig. 2B), the Cd_EX_ fraction decreased significantly by 80–75% under vermicompost and by 70–40% under compost compared to control sub-treatments. The Cd_CAR_ fraction decreased significantly by 60–40% and 25–30% under vermicompost and compost, respectively, compared to control sub-treatments. The Cd_OXD_ fraction decreased significantly by 15–30% under organic sub-treatments compared to the control. However, the organic-bound fraction significantly increased nine times under compost and vermicompost and 2–3 times when combined with NPK fertilizer compared to control sub-treatments. Its formation was more effective in compost than vermicompost when compared to the control. It decreased by 64–40% under organic amendments combined with NPK fertilizer compared to their single application (Cp and Vp). As a result, the residual fraction decreased significantly by 60–30% under vermicompost and by 25% under compost compared to control sub-treatments.

### Distribution of lead fractions in soil

**Figure 3:**
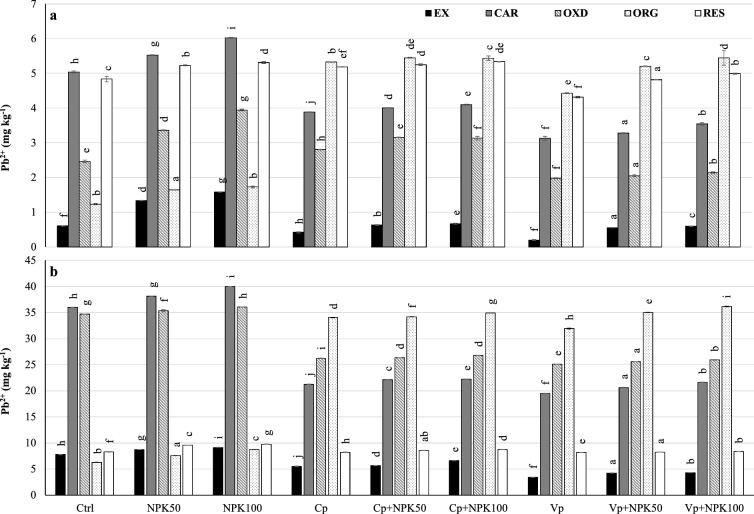
Distribution of Pb fractions in uncontaminated (**a**) and contaminated (**b**) soils. *Cd*_*EX*_ Exchangeable fraction, *Cd*_*CAR*_ Carbonate associated fraction, *Cd*_*OXD*_ Fe–Mn oxides bound fraction, *Cd*_*ORG*_ Organically bound fraction, *Cd*_*RES*_ Residual fraction, *Ctrl* Control, *NPK50* 50% of NPK dose, *NPK100* 100% of NPK dose, *Cp* Compost, *Cp+NPK50* Compost combined with 50% NPK, *Cp+NPK100* Compost combined with 100% NPK, *Vp* Vermicompost, *Vp+NPK50* Vermicompost combined with 50% NPK, *Vp+NPK100* Vermicompost combined with 100% NPK.

In uncontaminated soil (Fig. 3A), the Pb_EX_ fraction decreased significantly by 60% and 50% under vermicompost and compost, respectively, compared to control sub-treatments. The Pb_CAR_ fraction decreased significantly by 40% and 30% under vermicompost and compost respectively, compared to control sub-treatments. The Pb_OXD_ fraction decreased significantly by 20–40% under vermicompost sub-treatments compared to the control. This fraction significantly increased by 14% under compost but decreased significantly by 20% when combined with NPK fertilizer compared to the control sub-treatments. However, the Pb_ORG_ fraction increased 20–25 times under vermicompost sub-treatments and 21–33 times under compost compared to control sub-treatments. It decreased by 35% and 30% in soil amended with compost and combined with 50% and 100% NPK additions compared to compost alone. It decreased by 17% and 16% in soil amended with vermicompost and combined with 50% and 100% NPK additions compared to vermicompost alone. As a result, the residual fraction decreased significantly by 11%, 8%, and 6%, at Vp, Vp+NPK50, and Vp+NPK100 compared to control, NPK50, and NPK100, respectively, but only increased by 7% at Cp compared to control. Vermicompost was more effective than compost in reducing this fraction considering the formation of the immobilized fraction.

In contaminated soil (Fig. 3B), the exchangeable Pb_EX_ fraction decreased significantly by 55% and 30% under vermicompost and compost sub-treatments compared to the control. The Pb_CAR_ fraction significantly decreased by 46% and 42% under vermicompost and compost sub-treatments, respectively, compared to the control. The Pb_OXD_ fraction efficiently decreased by 28% at vermicompost and by 25% at compost alone or combined with NPK compared to control and NPK additions. However, the formed Pb_ORG_ fraction significantly increased 40 times under vermicompost and compost while increasing three times when combined with NPK fertilizer compared to the control. It showed a gradient decrease of 32–11% under organic treatments combined with 50% and 100% NPK rates, respectively, compared to their application alone. As a result, the residual fraction decreased significantly by 1.4%, 14%, and 15%, at Vp, Vp+NPK50, and Vp+NPK100, and by 1%, 10%, and 10% at Cp, Cp+NPK50, and Cp+NPK100 compared to control, NPK50, and NPK100, respectively.

### Crop yield assessment

Wheat crop yield was assessed by measuring plant height, the weight of 1000 grains, grain yield, straw yield, biological yield, harvest index, and NPK contents in grains (Supplementary Table S2). Contents of chromium, cadmium, and lead in wheat grains are also presented. Using Tukey´s HSD test, all parameters, except the harvest index (HI), varied significantly (*P*<0.05) within and between the studied treatments in both uncontaminated (small superscript letters) and contaminated (capital superscript letters) soils. The lack of significance in HI is due to the constant number of plant tillers computed for plant assessment. Significant improvement was observed in wheat crop yield parameters when the soil was enriched with organic amendments. The highest increase was in soils treated with vermicompost, followed by compost while the lowest assessment was in mineral NPK and control sub-treatments in both soils, with the highest improvement in uncontaminated.

## Discussion

An improvement in soil chemical conditions was observed in all organic treatments which enhanced in vermicompost-treated soils in both uncontaminated and contaminated soils. The overall changes in soil pH and organic reserve parameters under vermicompost and compost could be attributed to soil microbial metabolites (CO_2_ and organic acids) thus affecting soil-plant interaction^23–25^Nitrogen and phosphorus are more conserved by vermicompost than compost, not only because of their contents but due to composted metabolites^26^. However, organic carbon was higher under compost than vermicompost probably due to the high C/N ratio. SOC increased significantly with TN (r=0.717, *P*<0.01; r=0.383, *P*<0.05) and P_AV_ (r=0.606, *P*<0.01; r=0.413, *P*<0.05) in uncontaminated and contaminated soil, respectively. Soil alkalinity and organic reserves significantly improved along the organic treatments due to the low pH values mediated by organic and carboxylic acids added by compost and vermicompost^20^. Overall, soil amendments increased the nutrient stocks in both soils, and their stabilization mediated by soil microbial activities is significantly increased along the organic treatments^26^. Similarly, available phosphorus and potassium correlated significantly along organic treatments for uncontaminated (r=0.680, *P*<0.01) and contaminated soils (r=0.915, *P*<0.01) with the highest values under vermicompost-treated soils. Accordingly, SOC decreased significantly with soluble Na^+^ (r=−0.784, *P*<0.01 and r=−0.574, *P*<0.01), Ca^++^ (r=−0.611, *P*<0.01 and r=−0.530, *P*<0.01), and SAR (r=−0.513, *P*<0.01 and r=−0.623, *P*<0.01) for uncontaminated and contaminated soils, respectively, while soluble Mg^++^ decreased significantly (r=−0.669, *P*<0.01) in uncontaminated soil. A significant decrease in soluble Na was observed with the lowest values in vermicompost and compost compared to untreated and NPK-treated soils^20^. The presence of fulvic and humic acids (operationally defined as SOM extracts), added to soils through the organic amendments may have contributed to decrease the solubility of soil nutrients.

The highest values for wheat crop assessment parameters were observed in organic treatments combined with NPK additions. This was due to the relative improvement of soil micronutrient availability, along with the reduced bioavailability of transition metals in soil, which in turn promoted plant growth, improved general plant strength, and encouraged plant productivity. The soil’s biological properties were also improved as these organic additions acted as sources of plant nutrients. Similar results were obtained by Najar and Khan^27^ as vermicompost was a potential source of plant nutrients for sustainable tomato production.

Within the organic sub-treatments, vermicompost was more effective than compost in reducing the Cr availability in soil but re-increased when both were combined with NPK fertilizer in both soils. The immobilized Cr-organic fraction increased in organic sub-treatments compared to control sub-treatments in both soils. In uncontaminated soil, the higher immobilization was in compost than in vermicompost, however, both deteriorated with the increased NPK additions. In contaminated soil with higher Cr contents, vermicompost was more effective than compost in increasing the Cr_ORG_ fraction but regressed when both compost and vermicompost combined with NPK fertilizer. Similarly, Covelo et al.^28^ reported an increase in the Cr_ORG_ fraction under high Cr concentrations due to the high constant values ​​of stabilized Cr-organic complexes. The Cr forms were initially adsorbed by soil carbonates^28^ and redistributed along the organic treatments with a significant increase in immobilized fraction. This may indicate that the addition of organic amendments can enhance the Cr reduction rate to increase its temporal immobilization in soil^29^.

For cadmium, it can be observed that vermicompost was more effective than compost in reducing the available Cd fractions in both uncontaminated and contaminated soils, but both relapsed due to the NPK increase, except for the exchangeable fraction in compost. The formation of Cd-organic complexes was highly effective in compost than vermicompost compared to the control and mineral fertilizers^10,16^. This fraction decreased under the combined sub-treatments (organic and mineral) fertilizer compared to their alone application.

Vermicompost was more effective in decreasing the Pb availability than compost in both uncontaminated and contaminated soils, but only the exchangeable fraction, in contaminated soil, deteriorated when vermicompost was combined with NPK fertilizer. The Pb^2+^ solubility, in slightly to moderately contaminated soils, is controlled by its strong adsorption on Fe and Mn oxides and organic matter^30^. In highly contaminated soils, some Pb forms are stable enough to limit their solubility^31^. The higher efficiency for increasing the immobilized Pb_ORG_ fraction was in soils amended with compost than vermicompost in uncontaminated soil, however, both deteriorated when combined with mineral fertilizer. In contaminated soil, this fraction significantly increased under alone application of vermicompost and compost than combined with NPK fertilizer and the control.

The bioavailability of the studied metals was highly decreased under organic treatments in contaminated soils compared to their baseline levels in uncontaminated soils. Similarly, the immobilization in terms of increasing the formation of stabilized organic complexes highly occurred under organic treatments in contaminated soils and compared to the baseline levels of the studied metals in uncontaminated soils. Additionally, supplementary Table S2 shows the contents of chromium, cadmium, and lead in wheat grains in uncontaminated and contaminated soils. Their contents decreased significantly along the organic treatments with the lowest values in vermicompost followed by compost. In this regard, the decreased plant uptake for metals was attained due to the formation of stabilized organic compounds in soil and consequently decrease their translocations in wheat grains^29,32^. The formation of complex metalorganic associations in both solvated and solid phases, through complexity and specific adsorption, is an important mechanism responsible for hindering indigenous metal effects and diminishing their uptake by plants^33^.

The mobility factor of the studied metals was highly presented in control and NPK treatments in both soils (Supplementary Table S3). The mobility factor represents the relative amount of both the easily mobile (exchangeable, _EX_) and available (carbonate, _CAR_) fractions^34^ in the function of the total extracted metal fractions. The decreased mobility (%) of metals in organic treatments was presented as the differences between organic sub-treatments and their related control and NPK sub-treatments. The potential toxicity of metals in the soil is a function of their mobility and bioavailability due to the increasing tendency to enter readily into the food chain. Likewise, the bioaccumulation factor in wheat grains showed a similar trend confirming a decrease in plant uptake under compost and vermicompost due to the formation of organo-metallic complexes to reduce their availability^17^. A large decrease was observed in vermicompost and compost (underlined bold values), however, they were retracted with NPK additions combined with vermicompost and compost (bold values). A general observation showed the efficacy of vermicompost compared to compost in both soils. The mobility of chromium was highly decreased under uncontaminated soil while the mobility of cadmium and lead was highly decreased in contaminated soil due to the metal behavior in the soil as controlled by Ca contents^35^. Using Tukey´s HSD test, mobility, immobilization, and bioaccumulation parameters varied significantly (*P*<0.05) within and between the studied treatments in both uncontaminated (small superscript letters) and contaminated (capital superscript letters) soils.

Using Factor Analysis, all data for soil and plant analyses (varimax normalized) obtained from contaminated and uncontaminated soils were separately run (Supplementary Table S4). For the uncontaminated soil data, the first-three factors explained 92% of the total variance. The first factor explained 65% of the variance with high positive loadings >0.50 from soluble sodium, SAR, Pb_CAR_, Cr_CAR_, Cr_RES_, mobility of Cr, Cd, and Pb, Pb uptake in grains, and bioaccumulation factor of Cd and Pb. The positive factor scores contributed to these parameters were from the control (1.64), NPK50 (0.90), Cp+NPK0 (0.98), and Cp+NPK50 (0.20) treatments emphasizing the negative influences of mineral fertilizers to increase metals bioavailability and mobility and consequently their uptake by plant grains. Negative loadings > −0.50 were from EC, SOC, TN, P_AV_, K, Cr_ORG_, Pb_ORG_, plant height, wheat straw, grain yield, biological yield, and NPK in grains. The negative factor scores contributed to these parameters were from Vp+NPK100 (−1.43), Vp+NPK50 (−1.02), Cp+NPK100 (−0.58), Vp (−0.49), and NPK100 (−0.10) treatments indicating the contribution of vermicompost in increasing soil organic reserve, the immobilization of Cr and Pb, and consequently increase crop yield and quality (NPK contents in grains) compared to compost^5,6^.

The second factor explained 19% of the total variance with high positive loadings from soluble Na^+^, Ca^+2^, and Mg^+2^, available, Fe–Mn oxide, residual fractions of Cr, Cd, and Pb, the total content of Cd, and Pb in soil, potassium in grains, plant uptake of Cr, Cd, and Pb, and their bioaccumulation factor. The positive factor scores related to this factor were from NPK100 (1.97), NPK50 (0.89), and Cp+NPK100 (0.46) treatments emphasizing the increase in metal accumulation in plants due to the NPK addition. High negative loadings > −0.50 were from SOC and organic fractions of Cr, Cd, and Pb with the highest negative factor scores from Vp (−1.33), Cp (−0.89) emphasizing the immobilized fraction formation under compost and vermicompost. Intermediate negative factor scores were from Vp+NPK50 (−0.50) and Cp+NPK50 (−0.18) and positive scores from Cp+NPK100 (0.46) and Vp+NPK100 (0.02) corroborating the negative impacts of NPK, combined with Cp and Vp, on the immobilization of trace metals to increase their uptake by plants.

The third factor explained 8% of the total variance with high positive loadings > 0.50 from soil pH with the highest factor scores from control, NPK50, and NPK100. The high negative loadings > −0.50 were from SOC and organic fractions of Cr, Cd, and Pb, the residual fraction of Pb, the total content of Cr and Pb in soil, and harvest index with the highest factor scores from Cp, Cp+NPK50, and Cp+NPK100.

For data obtained from contaminated soil, the first-three factors explained 94% of the total variance. The first factor explained 65% of the variance with high positive loadings > 0.50 from soluble sodium, calcium, magnesium, available and residual fractions of Cr, Cd, and Pb, Fe–Mn oxide fraction of Cr and Pb, mobility and total content of Cr, Cd, and Pb in soil, uptake of Cr, Cd, and Pb by plant grains and their bioaccumulation factor. The highest factor scores for this factor were from NPK50 (1.5), NPK100 (0.73), Cp+NPK50 (0.39), and Cp+NPK100 (0.95) emphasizing the negative effects of mineral fertilizers to increase the availability of these metals and thus reducing their immobilization even when combined with compost. Along with the organic treatments, inhibition of Cd availability occurred which reduces the harmful effects of cadmium on growth. The highest positive factor scores from Cp+NPK50 (0.39) and Cp+NPK100 (0.95) with low pH values due to the organic additives, increasing metals solubility to become more available to plants^36^. The high negative loading was only from TN with the highest contribution from Vp (−1.68), Vp+NPK50 (−0.95), Vp+NPK100 (−0.03), and Cp (−0.53) with high available N forms.

The second factor explained 21% of the total variance with high positive loadings > 0.50 from soluble sodium, SAR, available and Fe–Mn oxide fractions of Pb, mobility factor of Cr and Pb, and harvest index. The relevant factor scores were from the control (1.26), NPK50 (0.79), and NPK100 (0.30). The high negative loadings were from EC, TN, P_AV_, K^+^, organic fraction of Cr, Cd, and Pb, Fe–Mn oxide fraction of Cd, plant height, wheat straw, grain yield, biological yield, and NPK uptake in grains. The highest factor scores were from Cp+NPK100 (−0.73), Cp+NPK50 (−0.06), Vp+NPK100 (−1.73), and Vp+NPK50 (−1.06). It can seem that plant height, wheat straw, grain yield, biological yield, and NPK uptake in grains variables moved to the second factor under contaminated treatments, in the second run, indicating that the increased sensitivity of plant yield to the addition of Pb, Cd, and Cr ions in soils as these parameters in parallel moved to the organic bound fractions of these metals and sodium adsorption capacity. The third factor explained 8% of the total variance with high positive loadings > 0.50 from soil pH, carbonates, and Fe–Mn oxide bound fractions of Cr and Pb and their mobility factor. Factor scores related to these parameters were from control (1.28), NPK50 (0.78), NPK100 (0.57), Vp+NPK50 (0.66), and Vp+NPK100 (0.37). The high negative loadings were from SOC and Pb_ORG_ with the highest contribution from Cp (−1.72), Cp+NPK50 (−1.08), Cp+NPK100 (−0.84), and vermicompost (−0.02).

**Figure 4:**
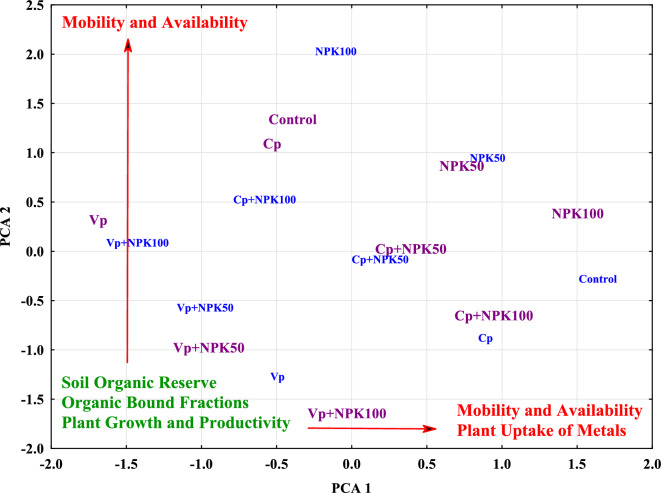
Relationship between the factor scores correspond to the first two PCAs run for both uncontaminated (labels with blue color) and contaminated (labels with violet color) soils.

The factor score values tested by the PCA (Principal Component Analysis) in Fig. 4 can display the contribution of each treatment to the relevant factor structures for uncontaminated (blue color) and contaminated (violet color) soils. The highest positive factor scores at the two factors were from soils only treated with NPK in both uncontaminated and contaminated soils correlated with the availability, mobility, bioavailability, and bioaccumulation factors of the studied metals. In addition, the lowest contribution for these parameters was from Vp, Vp+NPK50, and Vp+NPK100. The immediate contribution was recorded from Cp, Cp+NPK50, and Cp+NPK100. The combined compost and vermicompost with NPK resulted in a higher contribution to the above-stated parameters than their absolute application.

It can be also observed that soil organic reserve (Fig. 4) in terms of soil organic carbon, total nitrogen, soil phosphorus, available phosphorus and potassium, soluble cations, and organic fractions of Cr, Cd, and Pb, and total Cr contents resulted with the highest contribution from vermicompost and compost treatments compared to absolute NPK addition. This trend was observed in uncontaminated and contaminated soils by the highest negative factor scores related to the first and the second factor, respectively. As a result, the organic component amended with the added vermicompost and compost showed high importance for preserving organic components and mitigating hazard indices of Cr, Cd, and Pb^14,17^.

## Conclusions

The application of organic amendments (compost and vermicompost) recycled from agro-industrial residues improved the soil's chemical properties, which in turn reflected positively on the yield and quality of the wheat crop in both uncontaminated and contaminated soil. The highest improvement in soil chemical conditions was in vermicompost followed by compost while the lowest values were in NPK fertilizers (%50 and 100% NPK) and control in both soils. This improvement relapsed in vermicompost and compost treatments when combined with NPK additions, based on the applied NPK rates, in both soils. Wheat crops improved in both soils and showed the highest plant height, weight of 1000 grains, grain yield, straw yield, biological yield, and NPK contents of wheat grains in vermicompost and compost compared to NPK fertilizers and control treatments. An enhancement in wheat crop yield was observed in vermicompost and compost when combined with NPK additions.

This type of application in soils contaminated with chromium, cadmium, and lead reduced their availability in the soil and thus decreased their uptake by plants and their bioaccumulation in wheat grains. The metalorganic complexes formed by amended vermicompost and compost in soil transformed the soluble and/or exchangeable fractions of these metals thus reducing their availability to plants. The metal fractions redistributed in soil under organic amendments as ORG>OXD>CAR>RES>EX for chromium, ORG>RES>OXD>CAR>EX for cadmium, and ORG>OXD>CAR>RES>EX for lead, giving the massive amount for the organically bounded fractions. Due to complexation and adsorption or precipitation, the metal bonds with organic and oxides or in the residual fractions became not readily available for plants. Accordingly, these recycled amendments can mitigate the risk of contamination in the food chain by decreasing metal availability in soil and their uptake by the plant. Further research is needed to account for the importance of amendments in decreasing metal entry into the food chain and to assess their risks to human health. It is planning to produce a datasheet for the recycled agro-industrial residues to approve their efficiency as recommended soil amendments.

## Methods

### Experimental design

A field experiment was conducted during the season 2021/2022 in New Borg El-Arab City (30°53´33.17” N, 29°22´46.43” E), Alexandria governorate, Egypt. We confirm that experimental research and field studies on cultivated wheat plants, including the collection of plant material, comply with relevant institutional, national, and international guidelines and legislation. The field study was carried out on the experimental farm of SRTA-City, permissions were granted by the farm´s scientific counsel as our study complies with local and national regulations. Seeds of wheat “*Triticum aestivum* L.” (GIZA 171 cultivar) were purchased from the accredited national center for seeds at the Agricultural Research Centre, Ministry of Agriculture, Giza, Egypt. All agricultural practices for wheat crop production, including organic and mineral fertilization, were carried out according to the recommendations of the Egypt Ministry of Agriculture and Land Reclamation in 2013^37^. The carbonate content of the soil was detected by excess addition of 4M HCl and the released CO_2_ was quantified using the developed soil Royal Eijkelkamp calcimeter (Scheibler instrument, Eijkelkamp Soil & Water, Giesbeek, Netherlands) that meets the certified standard NEN-ISO 10693. Accurate measurements were in accordance with the Scheibler method which involves a determination of the carbonate content in the soil based on a volumetric method (measuring range: 0–>200 g kg^−1^ with a reading accuracy of 1 g kg^−1^).

The plot experiment was divided into two areas; uncontaminated soil, without any metal additions, and contaminated soil, treated with concentrations of CrCl_3_ (SIGMA-Aldrich Labrochemikalien GmbH, Riedstr.2, D-89555 Steinheim, Germany, 98.5%) (100 mg Cr per kg soil), PbCl_2_ (SIGMA-Aldrich Labrochemikalien GmbH, Riedstr.2, D-89555 Steinheim, Germany, 99.0%) (100 mg Pb per kg soil), and CdCl_2_ (SIGMA-Aldrich Labrochemikalien GmbH, Riedstr.2, D-89555 Steinheim, Germany, 99.0%) (3 mg Cd per kg soil) to reach the critical soil limits proposed by Alloway^38^and to exceed those limits encountered in our areas of alkaline soils^39^. Additionally, each soil was treated with either compost (Cp) or vermicompost (Vp) at a rate to reach 2% of soil organic matter, but the control. In both soils, compost and vermicompost, known as organic treatments, were sub-treated with NPK at different rates of 0% (control), 50% (NPK50), and 100% (NPK100)^37^. The 100% of NPK dose comprises 118 kg N per feddan of 33.5% N (NH_4_NO_3_), 29 kg P_2_O_5_ per feddan of 15.5% P_2_O_5_, and 59 kg K_2_O per feddan of 48% K_2_O. Phosphorus was added before cultivation while nitrogen and potassium were added in three batches: after germination, at the beginning of the vegetative growth stage, and tillering stage. Surface irrigation was applied at 4000 m^3^ ha^−1^ to provide water needs for the wheat crop and prevent any stress for metal translocation from soil to plant.

The Randomized Complete Block Design (RCBD) was used for the experimental design based on three factors: contamination (uncontaminated and contaminated soil), organic treatments (control, compost, and vermicompost), and mineral fertilizer levels (0, 50, and 100% NPK) with triplicate for each sub-treatment, giving a total of 54 sub-treatments. A total of eighteen sub-treatments were obtained, nine (n=27= 9×3 replicates) for the uncontaminated soil and nine (n=27= 9×3 replicates) for the contaminated soil (Supplementary Table S1). The term “control sub-treatments” is referring to the control, NPK50, and NPK100 sub-treatments. Accordingly, the term “compost sub-treatments” refers to Cp, Cp+NPK50, and Cp+NPK100 sub-treatments, and the term “vermicompost sub-treatments” refers to Vp, Vp+NPK50, and Vp+NPK100 sub-treatments. Each sub-treatment was conducted in squared (3×3 m) plots in triplicates.

### Soil analysis

After the experiment, soil samples from uncontaminated and contaminated soils were collected in triplicates from each sub-treatment (3×9×2=54 samples), air-dried, ground, and sieved at 2 mm for the subsequent soil analyses following the manual proposed by Ryan et al.^40^. Soil particle analysis was performed by using Robinson's pipette method (Eijkelkamp Agriresearch Equipment, Giesbeek, Netherlands). Soil pH and electrical conductivity (EC) were measured in 1:2.5 w/v and 1:1 w/v soil aqueous suspension, respectively. Soil organic carbon (SOC) was oxidized using the wet oxidation method. Total nitrogen (TN) was determined using the Kjeldahl method^40^. Soluble cations (Na^+^, K^+^, Ca^2+^, and Mg^2+^) and anions (SO_4_^2−^, Cl^−^, HCO_3_^−^) were determined from the saturated soil paste extracts^40^. Soluble Na^+^ and K^+^ were measured by the flame photometer (PG Instruments Ltd, Alma Park, Wibtoft, Leicestershire, England: FP902). The sodium adsorption ratio (SAR) was calculated as proposed by Robbins^41^.

1 $$SAR = \frac{{\text{Na }}}{{\sqrt {\frac{1}{2}\left( {{\text{Ca }} + {\text{Mg }}} \right)} }}$$

Soluble SO_4_^2−^ was determined by barium sulfate precipitation. Soluble Cl^−^ and HCO_3_^−^ were titrated with AgNO_3_ (SIGMA-Aldrich CHEMIE GmbH, P.O.1120–89555 Steinheim, Germany) and 0.01 N H_2_SO_4_ (SIGMA-Aldrich Laborechemikalien GmbH, D 30926 Seelze, Germany), respectively. The EDTA (SIGMA-Aldrich CHEMIE GmbH, Riedstr 2–89555 Steinheim, Germany) titration method was used to assess the soluble Ca^2+^ and Mg^2+^. Available phosphorus (P_AV_) was extracted with 0.5 M NaHCO_3_ (SIGMA-Aldrich CHEMIE GmbH, P.O.1120–89555 Steinheim, Germany) at pH 8.5 and measured by the PG Instruments Ltd T80 UV/VIS Spectrophotometer, Alma Park, Woodway Lane, Wibtoft, England^42^. Available potassium (K_AV_) was extracted with 1 N NH_4_OAc (PANREAC QUIMICA SA, E-08211 Castellar del valles, Barcelona, Spain) and measured by the flame photometer (PG Instruments Ltd, Alma Park, Wibtoft, Leicestershire, England: FP902).

Transition metals fractions were sequentially extracted^43,44^. Five fractions were determined for Cr^3+^, Cd^2+^, and Pb^2+^ as follows: the exchangeable fraction (_EX_) was extracted by 1 M MgCl_2_ (SIGMA-Aldrich CHEMIE GmbH, P.O.1120–89555 Steinheim, Germany) at pH 7 for 1 h at 25 °C; the fraction bound to carbonates (_CAR_) was extracted by 1 M CH_3_COONa (SIGMA-Aldrich CHEMIE GmbH, Riedstr 2 -89555 Steinheim, Germany) at pH 5 for 5 h at 25 °C; the fraction bound to Fe-Mn oxides (_OXD_) was extracted by 0.04 M NH_2_OH.HCl (SIGMA-Aldrich CHEMIE GmbH, D-30926 Seelze, Germany) in 25% acetic acid (SIGMA-Aldrich CHEMIE GmbH, P.O.1120 -89555 Steinheim, Germany) for 5 h at 96°C; the fraction bound to organic matter (_ORG_) was extracted by 30% H_2_O_2_ (PANREAC QUIMICA SA, E-08211 Castellar del valles, Barcelona, Spain) with 0.02 M HNO_3_ (SIGMA-Aldrich Laborchemikalien GmbH, D-30926 Seelze, Germany) of pH 2 for 5 h at 85°C followed by 3.2 M NH_4_OAc (SIGMA-Aldrich CHEMIE GmbH, Riedstr2, D-89555 Steinheim, Germany) in 20% HNO_3_(SIGMA-Aldrich Laborchemikalien GmbH, D-30926 Seelze, Germany); and the residual fraction (_RES_) was extracted by concentrated HNO_3_(SIGMA-Aldrich Laborchemikalien GmbH, D-30926 Seelze, Germany) for 2 h at 100°C. All fractions were filtered using Whatman No.1 filter paper (Whatman paper, Z240079) and then quantified with an Agilent 4100 Microwave Plasma-Atomic Emission Spectrometer (MP-AES) (Agilent Technologies, G8000A, Australia). The available fractions of each metal were assessed as the sum of exchangeable and carbonate-bound fractions^34^.

### Mobility assessment in soil

The mobility factor of Cr^3+^, Cd^2+^, and Pb^2+^ in soil was assessed to detect the relative amounts of the easily mobile and available fractions^34^ as follows:

2 $$Mobility\,factor\,\left( {\text{\% }} \right) = \frac{EX + CAR}{{EX + CAR + OXD + ORG + RES}}{ } \times 100$$

where _EX_ is the exchangeable fraction, _CAR_ is the carbonate-associated fraction, _OXD_ is the Fe–Mn oxides bound fraction, _ORG_ is the organically bound fraction, and _RES_ is the residual fraction.

### Chemical characterization of organic amendments

The recycled agro-industrial residues, by-products from food industries, were analyzed before their addition to soil^40^. Their pH, EC, organic carbon, total nitrogen, organic matter, and C/N ratio were 7.50, 2.16 dS m^−1^, 25.93%, 1.57%, 44.59%, and 16.51 for compost and were 7.98, 3.01 dS m^−1^, 36.41%, 1.89%, 62.63%, and 19.26 for vermicompost, respectively. Their NPK contents were 1.57%, 0.52%, and 1.02% for compost and 1.89%, 0.23%, and 0.41% for vermicompost, respectively. Their contents of Fe, Zn, Mn, and Cu were 4870.00, 35.40, 315.00, and 11.30 mg kg^−1^ for compost and 2081.00, 28.11, 127.00, and 7.22 mg kg^−1^ for vermicompost, respectively. Their contents of Cr, Cd, and Pb were 0.54, 0.23, and 5.93 mg kg^−1^ for compost and 0.32, 0.10, and 2.25 mg kg^−1^ for vermicompost, respectively.

### Chemical characterization of soil prior to the experiment

Moderate soil alkalinity (pH 8.39, 1:2.5 w/v) and salinity (EC of 2.69 dS m-1, 1:1 w/v) is observed. Contents of soluble cations such as Na^+^, K^+^, Ca^2+^, and Mg^+2^ were 7.97, 0.80, 7.15, and 0.82 meq L^−1^, respectively, while soluble anions such as Cl^−^, HCO_3_^−^, and SO_4_^2−^ were 6.12, 4.52, and 7.12 meq L^−1^, respectively. Total nitrogen (TN) and organic matter were 0.09% and 0.98%, respectively. Available soil phosphorus and potassium were 5.00 and 105.22 mg kg^−1^, respectively. Soil micronutrients such as Fe, Zn, Mn, and Cu were 4.81, 0.65, 7.74, and 0.85 mg kg^−1^, respectively. The contents of the studied three metals such as Cr, Cd, and Pb were 0.02, 0.01, and 0.66 mg kg^−1^, respectively.

### Plant analysis

After harvest (155 days), wheat plants (n=9) were randomly chosen from each sub-treatment (9×9×2=162 plant samples) to assess wheat crop yield by measuring plant height (cm), the weight of 1000-grains (g), wheat straw (t ha^−1^), and grain yield (t ha^−1^). The biological yield (t ha^−1^) was calculated as the sum of wheat straw and grain yield. The harvest index was presented as the ratio of grain yield to biological yield.

Wheat grains were washed with DH_2_O and oven-dried at 65 ℃ for 48 h. Grains were wet-digested using the H_2_SO_4_/H_2_O_2_ (SIGMA-Aldrich Laborechemikalien GmbH, D 30926 Seelze, Germany), mixture for the NPK and transition metals (Cr^3+^, Cd^2+^, and Pb^2+^) determinations following the manual proposed by Ryan et al.^40^.

### Bioaccumulation factor (BAF) in grains

BAF is the accumulation efficiency of each metal accumulated in plant grains^45^ and can be calculated using the following equation:

3 $$BAF = \frac{{Total\,Metal\,Content{ }_{grains} }}{{Total\,Metal\,Content_{{{ }soil}} }}$$

### Statistical analysis

Statistical analysis was run using STATISTICA 10 of StatSoft, Inc. (Tulsa, Oklahoma, USA)^46^. The two-factor ANOVA was tested to analyze the variability of all the studied soil and plant variables using mineral and organic amendments as the two categorical factors to check the significant variance of each data set obtained from uncontaminated and contaminated soils. Two runs of Principal Component Analysis were carried out for each data set obtained from uncontaminated and contaminated soils. The contribution of each sub-treatment (mineral×organic) to the related factor structure was calculated by factor scores.

## Supplementary Information

Supplementary Information.

## Data Availability

The datasets used and/or analyzed during the current study are included in this published article and more details can be available from the corresponding author upon reasonable request.
