# Supplementary material for: Immobilization of Cr3+, Cd2+, and Pb2+ added to calcareous soil amended with composted agro-industrial residues
Source: Sci Rep. 2023 May 20;13:8197. doi: 10.1038/s41598-023-35358-3 (PMC10199914; doi:10.1038/s41598-023-35358-3)
Supplement: Supplementary file 1 — Supplementary Information. [file 41598_2023_35358_MOESM1_ESM.docx]

**Supplementary Information**

**Immobilization of Cr^3+^, Cd^2+^, and Pb^2+^ added to calcareous soil amended with composted agro-industrial residues**

**Mai Khedr^1^, Mohamed Emran^1^*, Maria Gispert^2^, Mohamed Rashad^1^**

^1^ *Land and Water Technologies Department, Arid Lands Cultivation Research Institute (ALCRI), City of Scientific Research and Technological Applications (SRTA-City), 21934 New Borg El-Arab City, Alexandria, Egypt.*

^2^ *High Polytechnic School, University of Girona, C Maria Aurèlia Capmany, 61, Campus Montilivi, 17003 Girona, Spain.*

***Corresponding Author**

M. Emran, PhD

Email: [memran@srtacity.sci.eg](mailto:memran@srtacity.sci.eg) , [mohamed.emran@outlook.com](mailto:mohamed.emran@outlook.com)

**Supplementary Tables**

# **Supplementary Table S1.** Soil chemical characteristics of treated soils.

| **Treatments** | | **pH** | **EC**  **dS m^-1^** | **SOC**  **%** | **TN**  **%** | **P_AV_**  **mg kg^-1^** | **K_AV_**  **mg kg^-1^** | **Na^+^** | **K^+^** | **Ca^++^** | **Mg^++^** | **SAR** |
| --- | --- | --- | --- | --- | --- | --- | --- | --- | --- | --- | --- | --- |
|  |  |  |  |  |  |  |  | **mmol (+) L^−1^** | | | |  |
| Uncontaminated soil | Control | 8.20±0.01^f^ | 2.60±0.02^a^ | 0.39±0.00^a^ | 0.06±0.00^a^ | 36.14±0.01^a^ | 179.88±0.08^a^ | 8.53±0.01^g^ | 4.60±0.00^a^ | 7.74±0.00^g^ | 4.95±0.01^g^ | 3.39±0.00^i^ |
|  | NPK50 | 8.10±0.01^e^ | 3.04±0.01^b^ | 0.52±0.00^b^ | 0.07±0.00^a^ | 36.51±0.01^b^ | 245.08±0.08^b^ | 8.69±0.01^h^ | 6.27±0.00^b^ | 8.84±0.00^h^ | 6.00±0.03^i^ | 3.19±0.00^h^ |
|  | NPK100 | 8.02±0.01^d^ | 3.13±0.02^c^ | 0.60±0.01^c^ | 0.09±0.00^b^ | 36.71±0.01^c^ | 310.39±0.55^e^ | 8.70±0.00^h^ | 7.94±0.01^e^ | 9.00±0.01^i^ | 6.60±0.01^j^ | 3.12±0.00^d^ |
|  | Cp | 7.83±0.01^a^ | 3.28±0.02^d^ | 1.42±0.01^i^ | 0.11±0.00^c^ | 40.41±0.01^d^ | 275.05±0.04^c^ | 7.55±0.00^d^ | 7.30±0.00^c^ | 6.90±0.00^d^ | 4.04±0.00^c^ | 3.23±0.00^g^ |
|  | Cp+NPK50 | 7.96±0.05^bcd^ | 3.38±0.01^e^ | 1.32±0.01^g^ | 0.11±0.00^c^ | 40.58±0.01^e^ | 301.36±0.56^d^ | 7.57±0.00^e^ | 7.71±0.01^d^ | 7.04±0.00^e^ | 4.65±0.04^e^ | 3.13±0.01^e^ |
|  | Cp+NPK100 | 7.94±0.01^bc^ | 3.47±0.02^f^ | 1.36±0.01^h^ | 0.17±0.00^f^ | 40.70±0.01^f^ | 363.21±0.71^f^ | 7.60±0.01^f^ | 9.29±0.02^f^ | 7.42±0.00^f^ | 4.88±0.03^f^ | 3.07±0.01^c^ |
|  | Vp | 7.93±0.02^b^ | 3.29±0.01^d^ | 1.19±0.01^f^ | 0.12±0.01^d^ | 42.34±0.01^g^ | 440.31±0.46^i^ | 6.65±0.00^a^ | 11.26±0.01^i^ | 6.10±0.01^a^ | 2.69±0.02^a^ | 3.17±0.00^f^ |
|  | Vp+NPK50 | 8.00±0.01^cd^ | 3.46±0.02^f^ | 1.13±0.01^d^ | 0.16±0.00^e^ | 48.41±0.01^h^ | 370.35±0.59^g^ | 6.71±0.00^b^ | 9.47±0.02^g^ | 6.45±0.01^b^ | 3.96±0.01^b^ | 2.94±0.00^b^ |
|  | Vp+NPK100 | 7.96±0.05^bcd^ | 3.61±0.01^g^ | 1.17±0.00^e^ | 0.19±0.01^g^ | 50.11±0.04^i^ | 381.70±1.12^h^ | 6.73±0.00^c^ | 9.76±0.08^h^ | 6.85±0.04^c^ | 4.30±0.01^d^ | 2.85±0.01^a^ |
| Contaminated soil | Control | 8.07±0.02^E^ | 3.08±0.02^A^ | 0.30±0.01^A^ | 0.05±0.00^A^ | 35.47±0.02^A^ | 165.99±0.03^A^ | 8.70±0.00^G^ | 4.25±0.00^A^ | 8.94±0.01^F^ | 5.83±0.01^E^ | 3.20±0.00^G^ |
|  | NPK50 | 8.06±0.02^E^ | 3.34±0.01^C^ | 1.01±0.00^B^ | 0.06±0.00^A^ | 36.36±0.03^B^ | 190.88±0.51^B^ | 8.70±0.00^GH^ | 4.88±0.01^C^ | 9.19±0.06^G^ | 7.11±0.03^H^ | 3.05±0.01^F^ |
|  | NPK100 | 8.01±0.02^DE^ | 3.47 ±0.01^E^ | 1.05±0.00^C^ | 0.08±0.00^B^ | 36.58±0.02^C^ | 217.88±0.20^C^ | 8.70±0.00^H^ | 5.57±0.01^D^ | 9.00±0.13^F^ | 7.46±0.06^I^ | 3.03±0.01^F^ |
|  | Cp | 7.80±0.01^A^ | 3.28±0.01^B^ | 2.16±0.01^H^ | 0.10±0.00^C^ | 40.31±0.06^D^ | 230.63±0.08^D^ | 7.55±0.00^D^ | 5.90±0.00^E^ | 7.60±0.00^C^ | 5.12±0.01^D^ | 2.99±0.00^E^ |
|  | Cp+NPK50 | 7.90±0.04^B^ | 3.37 ±0.02^D^ | 1.45±0.01^F^ | 0.11±0.00^C^ | 40.49±0.01^E^ | 313.11±0.93^E^ | 7.56±0.00^E^ | 8.01±0.02^F^ | 8.29±0.02^D^ | 6.03±0.03^F^ | 2.83±0.00^C^ |
|  | Cp+NPK100 | 7.92±0.02^BC^ | 3.61±0.01^F^ | 1.71±0.02^G^ | 0.13±0.00^D^ | 40.56±0.03^E^ | 333.57±0.52^F^ | 7.61±0.00^F^ | 8.53±0.01^G^ | 8.55±0.00^E^ | 6.25±0.07^G^ | 2.80±0.00^B^ |
|  | Vp | 7.86±0.04^AB^ | 3.30±0.05^C^ | 1.43±0.02^E^ | 0.20±0.01^E^ | 42.41±0.08^F^ | 397.52±0.50^G^ | 6.65±0.00^A^ | 10.17±0.01^H^ | 6.64±0.06^A^ | 3.29±0.01^A^ | 2.99±0.01^E^ |
|  | Vp+NPK50 | 7.98±0.02^CD^ | 4.02 ±0.01^G^ | 1.24±0.01^D^ | 0.22±0.00^F^ | 48.20±0.01^G^ | 410.67±0.52^H^ | 6.71±0.00^B^ | 10.50±0.01^I^ | 7.15±0.03^B^ | 3.72±0.02^B^ | 2.88±0.00^D^ |
|  | Vp+NPK100 | 7.93±0.03^BC^ | 3.63 ±0.01^F^ | 1.25±0.01^D^ | 0.22±0.01^F^ | 49.80±0.07^H^ | 416.40±0.53^I^ | 6.73±0.00^C^ | 10.65±0.01^J^ | 7.58±0.01^C^ | 4.72±0.06^C^ | 2.71±0.01^A^ |

EC: Electrical conductivity; SOC: Soil organic carbon; TN: Total nitrogen; P_AV_: Available phosphorus; K_AV_: Available potassium; SAR: Sodium adsorption ratio; NPK50: 50% of NPK recommended dose; NPK100: 100% of NPK recommended dose; Cp: Compost; Cp+NPK50: Compost combined with 50% NPK; Cp+NPK100: Compost combined with 100% NPK; Vp: Vermicompost; Vp+NPK50: Vermicompost combined with 50% NPK; Vp+NPK100: Vermicompost combined with 100% NPK.

Different superscript letters indicate significant data variability within and between soil treatments in uncontaminated (small letters) and contaminated (capital letters) soils at α < 0.05 checked by the Tukey's HSD test. Same letters indicate no significant variability.

# **Supplementary Table S2.** **Wheat crop yield assessment and chemical characterizations of wheat grains.**

| **Treatments** | | **Plant height**  **cm** | **Weight of 1000-grains**  **g** | **Grain yield**  **t ha^-1^** | **Straw yield**  **t ha^-1^** | **Biological yield**  **t ha^-1^** | **Harvest index** | **N _GRAINS_**  **%** | **P _GRAINS_**  **%** | **K _GRAINS_**  **%** | **Cr^3+^ _GRAINS_** | **Cd^2+^ _GRAINS_** | **Pb^2+^ _GRAINS_** |
| --- | --- | --- | --- | --- | --- | --- | --- | --- | --- | --- | --- | --- | --- |
|  |  |  |  |  |  |  |  |  |  |  | **mg kg^-1^** | | |
| Uncontaminated soil | Control | 48.82±0.02^a^ | 20.11±0.01^a^ | 1.53±0.00^a^ | 1.00±0.00^a^ | 2.52±0.00^a^ | 0.60±0.00^a^ | 1.3.6±0.01^a^ | 0.14±0.00^a^ | 0.45±0.00^c^ | 0.17±0.00^f^ | 0.05±0.00^e^ | 1.02±0.02^g^ |
|  | NPK50 | 53.67±0.01^b^ | 20.52±0.01^b^ | 1.64±0.00^b^ | 1.07±0.00^b^ | 2.70±0.00^b^ | 0.60±0.00^a^ | 2.38±0.01^b^ | 0.16±0.01^d^ | 0.53±0.00^a^ | 0.21±0.00^h^ | 0.07±0.00^f^ | 1.15±0.01^h^ |
|  | NPK100 | 59.50±0.02^c^ | 21.37 ±0.01^c^ | 1.81±0.00^c^ | 1.18±0.00^c^ | 2.98±0.00^c^ | 0.60±0.00^a^ | 2.95±0.04^c^ | 0.26 ±0.00^f^ | 0.58±0.01^h^ | 0.24±0.01^i^ | 0.08±0.01^g^ | 1.21±0.01^i^ |
|  | Cp | 60.00±0.40^d^ | 33.80±0.02^d^ | 2.15±0.00^d^ | 1.40±0.00^d^ | 3.55±0.00^d^ | 0.60±0.00^a^ | 3.17±0.01^d^ | 0.14±0.01^a^ | 0.41±0.00^b^ | 0.14±0.00^c^ | 0.02±0.00^a^ | 0.51±0.00^d^ |
|  | Cp+NPK50 | 69.52 ±0.11^f^ | 36.35±0.03^e^ | 2.56±0.00^e^ | 1.67±0.00^e^ | 4.23±0.00^e^ | 0.60±0.00^a^ | 3.38±0.02^e^ | 0.19±0.00^b^ | 0.51±0.01^e^ | 0.16±0.00^e^ | 0.03±0.00^b^ | 0.64±0.01^e^ |
|  | Cp+NPK100 | 76.10 ±0.09^h^ | 42.00±0.01^f^ | 2.85±0.00^f^ | 1.86±0.00^g^ | 4.71±0.00^g^ | 0.60±0.00^a^ | 3.65±0.01^f^ | 0.19±0.00^b^ | 0.53±0.00^a^ | 0.19±0.00^g^ | 0.04±0.00^d^ | 0.96±0.01^f^ |
|  | Vp | 65.00 ±0.01^e^ | 45.13±0.02^g^ | 2.80±0.00^g^ | 1.83±0.00^f^ | 4.62±0.00^f^ | 0.60±0.00^a^ | 3.72±0.03^g^ | 0.15±0.01^c^ | 0.49±0.01^d^ | 0.11±0.00^a^ | 0.00±0.00^c^ | 0.22±0.00^a^ |
|  | Vp+NPK50 | 72.50 ±0.63^g^ | 47.00±0.13^h^ | 2.95±0.00^h^ | 1.93±0.00^h^ | 4.88±0.00^h^ | 0.60±0.00^a^ | 4.67±0.02^h^ | 0.22±0.01^e^ | 0.54±0.01^f^ | 0.13±0.01^b^ | 0.02±0.00^a^ | 0.36±0.00^b^ |
|  | Vp+NPK100 | 79.00 ±0.00^i^ | 48.99±0.06^i^ | 2.98±0.00^i^ | 1.94±0.00^i^ | 4.92±0.00^i^ | 0.60±0.00^a^ | 4.84±0.03^i^ | 0.30 ±0.00^g^ | 0.55±0.01^g^ | 0.15±0.00^d^ | 0.03±0.00^b^ | 0.45±0.02^c^ |
| Contaminated soil | Control | 35.67±0.04^A^ | 24.06±0.03^A^ | 0.88±0.00^A^ | 0.56±0.00^A^ | 1.43±0.00^A^ | 0.61±0.00^A^ | 0.16±0.01^A^ | 0.09±0.00^A^ | 0.27±0.00^A^ | 0.43±0.02^F^ | 0.05±0.00^A^ | 1.18±0.01^B^ |
|  | NPK50 | 38.16±0.02^B^ | 25.03±0.02^B^ | 0.95±0.00^B^ | 0.60±0.00^B^ | 1.55±0.00^B^ | 0.61±0.00^A^ | 0.23±0.00^B^ | 0.10±0.00^B^ | 0.26±0.00^B^ | 0.61±0.01^H^ | 0.07±0.00^B^ | 1.34±0.00^G^ |
|  | NPK100 | 39.22±0.01^C^ | 26.09 ±0.01^D^ | 0.95±0.00^C^ | 0.60±0.00^B^ | 1.55±0.00^B^ | 0.61±0.00^A^ | 0.29±0.00^C^ | 0.14±0.00^C^ | 0.28±0.00^D^ | 0.74±0.00^I^ | 0.10±0.01^G^ | 1.62±0.04^H^ |
|  | Cp | 45.33 ±0.20^D^ | 26.00 ±0.06^C^ | 1.02±0.00^D^ | 0.64±0.00^C^ | 1.66±0.00^C^ | 0.61±0.00^A^ | 1.09±0.01^D^ | 0.10±0.00^D^ | 0.28±0.00^C^ | 0.21±0.01^B^ | 0.05±0.00^A^ | 0.89±0.02^A^ |
|  | Cp+NPK50 | 49.67±0.39^F^ | 26.67 ±0.04^E^ | 1.19±0.00^F^ | 0.75±0.00^D^ | 1.95±0.00^E^ | 0.61±0.00^A^ | 1.84±0.01^F^ | 0.15±0.00^F^ | 0.35±0.00^E^ | 0.38±0.00^D^ | 0.07±0.00^B^ | 1.03±0.02^E^ |
|  | Cp+NPK100 | 49.87 ±0.01^G^ | 30.00±0.02^F^ | 1.33±0.00^G^ | 0.84±0.00^E^ | 2.17±0.00^F^ | 0.61±0.00^A^ | 1.96±0.01^G^ | 0.18±0.00^G^ | 0.40±0.00^F^ | 0.45±0.01^G^ | 0.08±0.00F | 1.20±0.01^B^ |
|  | Vp | 47.33±0.17^E^ | 30.15 ±0.08^G^ | 1.19±0.00^E^ | 0.75±0.00^D^ | 1.94±0.00^D^ | 0.61±0.00^A^ | 1.28±0.01^E^ | 0.12±0.00^E^ | 0.30±0.00^G^ | 0.19±0.00^A^ | 0.03±0.01^C^ | 0.64±0.01^C^ |
|  | Vp+NPK50 | 50.67±0.00^H^ | 33.56 ±0.04^H^ | 1.52±0.00^H^ | 0.96±0.00^F^ | 2.48±0.00^G^ | 0.61±0.00^A^ | 2.10±0.01^I^ | 0.18±0.00^H^ | 0.39±0.00^H^ | 0.23±0.00^C^ | 0.04±0.02^D^ | 0.71±0.00^D^ |
|  | Vp+NPK100 | 51.67±0.03^I^ | 32.00±0.00^I^ | 1.74±0.00^I^ | 1.10±0.00^G^ | 2.84±0.00^H^ | 0.61±0.00^A^ | 2.13±0.01^H^ | 0.19±0.00^I^ | 0.46±0.00I | 0.39±0.01^E^ | 0.06±0.00^E^ | 0.91±0.01^A^ |

NPK50: 50% of NPK recommended dose; NPK100: 100% of NPK recommended dose; Cp: Compost; Cp+NPK50: Compost combined with 50% NPK; Cp+NPK100: Compost combined with 100% NPK; Vp: Vermicompost; Vp+NPK50: Vermicompost combined with 50% NPK; Vp+NPK100: Vermicompost combined with 100% NPK.

Different superscript letters indicate significant data variability within and between soil treatments in uncontaminated (small letters) and contaminated (capital letters) soils at α < 0.05 checked by the Tukey's HSD test. Same letters indicate no significant variability.

# **Supplementary Table S3.** Mobility, bioaccumulation factor, and immobilization of organic treatments of chromium, cadmium, and lead.

| **Treatments** | | **Chromium** | | **Mobility decrease of Cr^3+^ in organic treatments**  **%** | **Cadmium** | | **Mobility decrease of Cd^2+^ in organic treatments**  **%** | **Lead** | | **Mobility decrease of Pb^2+^ in organic treatments**  **%** |
| --- | --- | --- | --- | --- | --- | --- | --- | --- | --- | --- |
|  |  | **Mobility**  **%** | **BAF ratio in plant** |  | **Mobility**  **%** | **BAF ratio in plant** |  | **Mobility**  **%** | **BAF ratio in plant** |  |
| Uncontaminated soil | Control | 24.92±0.01^e^ | 0.0134±0.0002^e^ |  | 35.65±0.52^e^ | 0.0301±0.0006^f^ |  | 39.79±0.14^g^ | 0.0717±0.0011^i^ |  |
|  | NPK50 | 24.95±0.15^e^ | 0.0160±0.0001^f^ |  | 34.90±1.11^e^ | 0.0307±0.0007^f^ |  | 40.11±0.11^g^ | 0.0671±0.0007^h^ |  |
|  | NPK100 | 25.54±0.03^f^ | 0.0180±0.0007^g^ |  | 37.33±0.11^f^ | 0.0247±0.0014^e^ |  | 40.91±0.04^h^ | 0.0651±0.0005^g^ |  |
|  | Cp | 1.13±0.04^b^ | 0.0090±0.0002^a^ | **-95** | 22.99±0.29^b^ | 0.0127±0.0012^bc^ | **-36** | 24.44±0.06^c^ | 0.0292±0.0002^d^ | **-39** |
|  | Cp+NPK50 | 1.65±0.00^c^ | 0.0103±0.0000^c^ | **-93** | 23.49±0.31^b^ | 0.0166±0.0010^d^ | **-33** | 25.07±0.03^d^ | 0.0344±0.0004^e^ | **-38** |
|  | Cp+NPK100 | 2.09±0.01^d^ | 0.0113±0.0001^d^ | **-92** | 28.98±0.23^c^ | 0.0166±0.0005^d^ | **-22** | 25.54±0.04^e^ | 0.0517±0.0008^f^ | **-38** |
|  | Vp | 0.92±0.08^a^ | 0.0084±0.0004^a^ | **-96** | 19.84±0.41^a^ | 0.0015±0.0010^a^ | **-44** | 23.69±0.25^a^ | 0.0153±0.0004^a^ | **-40** |
|  | Vp+NPK50 | 1.51±0.05^c^ | 0.0092±0.0002^ab^ | **-94** | 23.14±0.02^b^ | 0.0113±0.0009^b^ | **-34** | 24.11±0.05^b^ | 0.0224±0.0003^b^ | **-40** |
|  | Vp+NPK100 | 2.06±0.01^d^ | 0.0100±0.0000^bc^ | **-92** | 23.32±0.16^b^ | 0.0140±0.0006^c^ | **-38** | 24.76±0.14^cd^ | 0.0267±0.0002^c^ | **-39** |
| Contaminated soil | Control | 37.33±0.01^H^ | 0.0040±0.0001^D^ |  | 45.60±0.25^G^ | 0.0142±0.0003^C^ |  | 47.03±0.02^H^ | 0.0126±0.0001^E^ |  |
|  | NPK50 | 40.67±0.01^I^ | 0.0052±0.0000^E^ |  | 51.09±0.23^H^ | 0.0161±0.0003^D^ |  | 47.15±0.09^I^ | 0.0135±0.0001^F^ |  |
|  | NPK_100 | 41.31±0.01^J^ | 0.0061±0.0001^F^ |  | 51.25±0.16^H^ | 0.0192±0.0010^F^ |  | 47.36±0.01^J^ | 0.0157±0.0003^G^ |  |
|  | Cp | 26.54±0.04^D^ | 0.0024±0.0001^B^ | **-29** | 25.13±0.27^D^ | 0.0168±0.0001^DE^ | **-45** | 28.10±0.01^D^ | 0.0093±0.0002^B^ | **-40** |
|  | Cp+NPK50 | 27.62±0.06^E^ | 0.0036±0.0000^C^ | **-32** | 35.09±0.00^E^ | 0.0186±0.0002^F^ | **-31** | 28.68±0.02^E^ | 0.0106±0.0002^C^ | **-39** |
|  | Cp+NPK100 | 28.93±0.01^F^ | 0.0041±0.0001^D^ | **-30** | 37.01±0.11^F^ | 0.0180±0.0007^EF^ | **-28** | 29.07±0.01^F^ | 0.0120±0.0001^D^ | **-39** |
|  | Vp | 24.93±0.00^A^ | 0.0021±0.0000^A^ | **-33** | 18.88±0.14^A^ | 0.0116±0.0009^AB^ | **-59** | 26.01±0.00^A^ | 0.0072±0.0001^A^ | **-45** |
|  | Vp+NPK50 | 25.07±0.08^B^ | 0.0023±0.0000^AB^ | **-38** | 21.04±0.06^B^ | 0.0110±0.0008^A^ | **-59** | 26.54±0.03^B^ | 0.0076±0.0000^A^ | **-44** |
|  | Vp+NPK100 | 25.81±0.06^C^ | 0.0037±0.0000^C^ | **-38** | 24.15±0.13^C^ | 0.0129±0.0002^BC^ | **-53** | 26.94±0.01^C^ | 0.0094±0.0001^B^ | **-43** |

NPK50: 50% of NPK recommended dose; NPK100: 100% of NPK recommended dose; Cp: Compost; Cp+NPK50: Compost combined with 50% NPK; Cp+NPK100: Compost combined with 100% NPK; Vp: Vermicompost; Vp+NPK50: Vermicompost combined with 50% NPK; Vp+NPK100: Vermicompost combined with 100% NPK; BAF: Bioaccumulation factor.

Different superscript letters indicate significant data variability within and between soil treatments in uncontaminated (small letters) and contaminated (capital letters) soils at α < 0.05 checked by the Tukey's HSD test. Same letters indicate no significant variability.

Underlined bold values indicate the highest decrease observed in vermicompost and compost compared to control.

Bold values indicate the decreased percentage under vermicompost and compost combined with NPK compared to the absolute NPK fertilizer

# **Supplementary Table S4.** The first three-factor structures from Factor Analysis run for the uncontaminated and contaminated soil.

| **Variables** | **Uncontaminated soil** | | | **Contaminated soil** | | |
| --- | --- | --- | --- | --- | --- | --- |
|  | **Factor 1** | **Factor 2** | **Factor 3** | **Factor 1** | **Factor 2** | **Factor 3** |
| pH | 0.44 | 0.34 | 0.64* | 0.36 | 0.16 | 0.87* |
| EC | -0.86* | -0.13 | -0.46 | 0.01 | -0.82* | 0.06 |
| SOC | -0.52* | -0.51* | -0.67* | -0.09 | -0.20 | -0.90* |
| TN | -0.89* | -0.20 | -0.23 | -0.54* | -0.82* | -0.05 |
| P_AV_ | -0.83* | -0.39 | 0.00 | -0.44 | -0.86* | -0.08 |
| Na^+^ | 0.73* | 0.68* | 0.05 | 0.65* | 0.69* | 0.31 |
| K^+^ | -0.89* | -0.24 | 0.02 | -0.44 | -0.85* | -0.14 |
| Ca^2+^ | 0.45 | 0.88* | 0.07 | 0.83* | 0.41 | 0.27 |
| Mg^2+^ | 0.33 | 0.93* | -0.05 | 0.93* | 0.36 | 0.07 |
| SAR | 0.93* | -0.13 | 0.12 | -0.09 | 0.89* | 0.42 |
| Cr _EX_ | 0.45 | 0.82* | 0.36 | 0.78* | 0.38 | 0.37 |
| Cr _CAR_ | 0.58* | 0.67* | 0.46 | 0.70* | 0.42 | 0.58* |
| Cr _OXD_ | 0.31 | 0.84* | -0.14 | 0.59* | -0.10 | 0.71* |
| Cr _ORG_ | -0.60* | -0.57* | -0.56* | -0.38 | -0.80* | -0.45 |
| Cr _RES_ | 0.63* | 0.74* | 0.21 | 0.81* | 0.43 | -0.02 |
| Cd _EX_ | -0.07 | 0.79* | -0.12 | 0.84* | 0.34 | 0.40 |
| Cd _CAR_ | 0.23 | 0.94* | 0.18 | 0.96* | 0.09 | 0.25 |
| Cd _OXD_ | 0.25 | 0.94* | 0.11 | 0.33 | -0.69* | 0.48 |
| Cd _ORG_ | -0.78* | -0.29 | -0.52* | -0.34 | -0.83* | -0.43 |
| Cd _RES_ | 0.25 | 0.95* | 0.12 | 0.81* | 0.32 | 0.38 |
| Pb _EX_ | 0.18 | 0.95* | 0.08 | 0.78* | 0.53* | 0.31 |
| Pb _CAR_ | 0.56* | 0.82* | 0.14 | 0.58* | 0.53* | 0.60* |
| Pb _OXD_ | 0.34 | 0.82* | -0.38 | 0.56* | 0.57* | 0.59* |
| Pb _ORG_ | -0.62* | -0.51* | -0.59* | -0.40 | -0.64* | -0.65* |
| Pb _RES_ | 0.18 | 0.68* | -0.68* | 0.82* | 0.21 | 0.26 |
| Cr _SOIL_ | -0.33 | -0.01 | -0.89* | 0.88* | 0.04 | 0.47 |
| Cd _SOIL_ | -0.09 | 0.97* | -0.09 | 0.90* | -0.33 | 0.24 |
| Pb _SOIL_ | -0.13 | 0.60* | -0.79* | 0.97* | -0.05 | -0.01 |
| Cr _Mobility_ | 0.59* | 0.65* | 0.47 | 0.64* | 0.55* | 0.53* |
| Cd _Mobility_ | 0.52* | 0.78* | 0.28 | 0.78* | 0.49 | 0.37 |
| Pb _Mobility_ | 0.59* | 0.68* | 0.43 | 0.53* | 0.60* | 0.60* |
| Plant height | -0.89* | -0.12 | -0.37 | -0.27 | -0.82* | -0.49 |
| Wheat straw | -0.87* | -0.41 | -0.22 | -0.22 | -0.96* | -0.06 |
| Grain yield | -0.87* | -0.41 | -0.22 | -0.22 | -0.96* | -0.06 |
| Biological yield | -0.87* | -0.41 | -0.22 | -0.22 | -0.96* | -0.06 |
| Harvest index | -0.05 | 0.06 | -0.58* | -0.13 | 0.64* | 0.18 |
| N _GRAINS_ | -0.94* | -0.21 | -0.18 | -0.25 | -0.84* | -0.42 |
| P _GRAINS_ | -0.73* | 0.49 | 0.05 | 0.15 | -0.96* | -0.08 |
| K _GRAINS_ | -0.63* | 0.70* | 0.19 | -0.03 | -0.96* | -0.12 |
| Cr _GRAINS_ | 0.26 | 0.96* | -0.06 | 0.88* | 0.16 | 0.40 |
| Cd _GRAINS_ | 0.35 | 0.93* | 0.11 | 0.98* | -0.01 | -0.08 |
| Pb _GRAINS_ | 0.51* | 0.82* | 0.01 | 0.90* | 0.32 | 0.25 |
| Cr _BAF_-_GRAINS_ | 0.38 | 0.89* | 0.24 | 0.89* | 0.16 | 0.38 |
| Cd _BAF-GRAINS_ | 0.62* | 0.67* | 0.12 | 0.83* | 0.31 | -0.43 |
| Pb _BAF-GRAINS_ | 0.61* | 0.70* | 0.16 | 0.87* | 0.38 | 0.28 |
| Variance (%) | **65** | **19** | **8** | **65** | **21** | **8** |

TN: Total nitrogen; P_AV_: Available phosphorus; _EX_: exchangeable fraction; _CAR_: Carbonates associated fraction; _OXD_: Fe-Mn oxides bound fraction; _ORG_: Organically bound fraction; _RES_: Residual fraction; _BAF_-_GRAINS_: Bioaccumulation factor of metal in grains. *: Factor loadings > 0.50.
